# Editorial: Pathogenic microbiology in West Africa

**DOI:** 10.3389/fmicb.2023.1255032

**Published:** 2023-07-24

**Authors:** Shuchao Wang, Foday Sahr, Mohamed Boie Jalloh, Canjun Zheng, Zhiqiang Mi

**Affiliations:** ^1^Changchun Veterinary Research Institute, Chinese Academy of Agricultural Sciences, Changchun, China; ^2^College of Medicine and Allied Health Sciences, University of Sierra Leone, Freetown, Sierra Leone; ^3^Chinese Center for Disease Control and Prevention, Beijing, China; ^4^Beijing Institute of Microbiology and Epidemiology, Beijing, China

**Keywords:** pathogenic microbiology, West Africa, *Escherichia Coli*, adenovirus, brucellosis, monkeypox (mpox)

West Africa, which consists of 16 countries and one dependency, is geographically situated with the Atlantic Ocean to the west and south, Middle Africa to the east, and North Africa to the north. Over the past five decades, West Africa has consistently faced a recurring wave of newly emerging and resurging infectious diseases. These include, but are not limited to, Marburg, meningitis, cholera, measles, yellow fever, Lassa fever, Ebola, COVID-19, and most recently, monkeypox (Nkengasong and Tessema, 2020). Moreover, the high occurrence of endemic diseases, such as malaria, HIV/AIDS, hepatitis, tuberculosis, and typhoid, has had a significant and negative effect on the overall state of public health within this specific region (Endy, 2020). Among all the continents, Africa bears the largest burden of these diseases and contributes significantly to the estimated 10 million annual deaths attributed to infectious diseases.

The reasons for the frequent emergence of novel diseases and the inability of health systems to effectively control localized epidemics of infectious diseases are multifactorial and have been extensively discussed in the literature (Nkengasong and Tessema, 2020; Nachega et al., 2023). One notable deficiency lies in the inadequacy of local capacity construction in laboratory medicine, particularly in the field of microbiological research (Nkengasong et al., 2018). The scarcity and outdatedness of fundamental data regarding infectious diseases and their associated pathogens in West Africa, particularly for diseases that are not deemed high priority, is a clear indication of this issue. For instance, despite Sierra Leone being severely affected by rabies, epidemiological and microbiological data on this disease in the country are rarely available from public sources (Talbi et al., 2009). In view of the prevailing circumstances, the present Research Topic endeavors to gather contributions from local stakeholders and reliable partners, with a specific focus on epidemiological and microbiological investigations encompassing diverse pathogens.

The ongoing outbreak of mpox, previously known as monkeypox, has been officially declared a Public Health Emergency of International Concern by the World Health Organization. This particular outbreak has been linked to the outbreak in Nigeria during the period of 2017–2018 (Isidro et al., 2022). Between January and August in 2022, a total of 220 confirmed cases and 4 deaths were documented during the new outbreak. Stephen et al. conducted an analysis of the epidemiological profiles of suspected patients in an eastern state of Nigeria, Adamawa, an area where outbreaks of mpox had not been previously reported. In contrast to other areas experiencing outbreaks of mpox, which primarily affected gay and bisexual adults, this study discovered that all 33 suspected patients in Adamawa were heterosexual and 18 (54.5%) were under the age of 19. Among the 24 confirmed cases, 15 (62.5%) were individuals who were either children or adolescents. This finding, when taken into consideration along with the observation of a low incidence of mpox alone, a high incidence of simultaneous mpox and varicella infections, and a high incidence of varicella alone, emphasizes the need for enhanced differential diagnostic techniques to distinguish between mpox and varicella in children and adolescents. This is especially crucial in nations with limited resources. The study also raised inquiries regarding the biological plausibility of the susceptibility to co-infection of these two diseases. Grose sided with the Opinion that varicella might precede mpox and play a role in the subsequent acquisition of mpox. This conclusion was drawn from an analysis of epidemiological data, pathogenic characteristics, and the incubation periods of both diseases. Furthermore, the clustering patterns of varicella during the outbreak provided additional evidence to support this argument. Nevertheless, additional cohort studies were recommended to corroborate this hypothesis.

Adenovirus type 7 (HAdV7) is considered one of the most pathogenic adenoviruses, capable of inducing severe complications, especially in immunocompromised individuals. It has been reported to be common in Asia and America, whereas the level of awareness regarding this disease remains relatively low in West Africa. Wang et al. developed an innovative approach for testing neutralizing antibodies (NA) against HAdV7 by ultilizing a chimeric HAdV7 expressing luciferase as a reporter virus and analyzed both the prevalence and titer of HAdV7 NA in populations from China and Sierra Leone. The study revealed that approximately 60% of the Chinese individuals have been affected by HAdV7 infection, which represented a significant increase compared to the historical positive rate of 10–30%. This finding suggested a notable surge in recent HAdV7 infections in China. In Sierra Leone, the prevalence of the disease was found to be higher than that in China, thereby highlighting the need to incorporate this condition into clinical settings.

Antimicrobial resistance has emerged as a pressing global issue; nevertheless, there is a significant lack of available data pertaining to this issue in West Africa. Hoffmann et al. observed that the *bla*_*CTX*−*M*−15_ gene associated with resistance against third-generation cephalosporins was highly prevalent in *Escherichia Coli* strains isolated from stool samples of European soldiers experiencing diarrhea during their deployment in Mali, West Africa. Through the molecular analysis of the full genome, it was also discovered that there was significant genetic variability among the isolates, a wide range of resistance genes encoded by plasmids and chromosomes, and the intricate nature of mobile genetic elements associated with antimicrobial resistance genes. These findings collectively indicated a grave concern regarding the issue of antimicrobial resistance in this specific region.

Brucellosis is a widespread zoonosis considerably affecting the ruminant feeding industry, which holds a crucial position in the economy of West Africa. Liu et al. conducted a comprehensive phylogenetic analysis of the pathogen *Brucella abortus* in West Africa. This analysis was conducted by employing various molecular tools and gathering relevant molecular data from publicly accessible repositories. The findings revealed a consistent presence of *Brucella abortus* across West Africa and its neighboring countries. Furthermore, it was also concluded that the majority of local cases in West Africa were caused by native strains, while a portion of the cases could be traced back to introduced lineages.

In summary, the studies in this Research Topic have expanded our understanding of *pathogenic microbiology in West Africa*, especially underscoring the importance of recognizing and tackling infectious diseases that have typically been overlooked in this region. These findings emphasize the need to incorporate these neglected diseases into clinical and public health practices for effective management and prevention.

## Author contributions

SW, FS, CZ, and ZM are guest editors of this Research Topic. SW drafted this Editorial. FS, MJ, CZ, and ZM reviewed and revised the manuscript. All authors contributed to the article and approved the submitted version.
